# Acute Aortic Dissection during Pregnancy: Hideous Clinical Quandaries with Young Lives on the Line—The Role of Hereditary Genetic Syndromes

**DOI:** 10.3390/jcm13164901

**Published:** 2024-08-20

**Authors:** Josephina Haunschild, Paulina Wiktorowska, Sandra Eifert, Holger Stepan, Ingo Dähnert, Michael A. Borger, Christian D. Etz

**Affiliations:** 1 Department of Cardiac Surgery, Rostock Heart Center, University Medical Center Rostock, 18057 Rostock, Germany; christian.etz@med.uni-rostock.de; 2 Department of Internal Medicine I, Division of Cardiology, Angiology and Intensive Medical Care, Friedrich-Schiller-University, University Hospital Jena, 07743 Jena, Germany; paulina.wiktorowska@med.uni-jena.de; 3 University Department of Cardiac Surgery, Leipzig Heart Center, 04289 Leipzig, Germany; sandra.eifert@helios-gesundheit.de (S.E.); michael.borger@helios-gesundheit.de (M.A.B.); 4 Department of Obstetrics, University Hospital Leipzig, 04103 Leipzig, Germany; holger.stephan@medizin.uni-leipzig.de; 5 Department of Pediatric Cardiology, Leipzig Heart Center, 04289 Leipzig, Germany; ingo.daehnert@helios-gesundheit.de

**Keywords:** acute aortic dissection, pregnancy, genetic syndrome

## Abstract

**Objective:** Acute aortic dissection is a rare but frequently fatal aortic catastrophe with high morbidity and mortality. Especially in pregnant patients, acute dissection is often misdiagnosed putting two lives on the line. Due to its scarcity, only case reports have been reported. The aim of this study is to analyze the time of aortic dissection during the course of pregnancy and the outcome of emergency surgery in pregnant women with and without hereditary connective tissue disorder. **Methods:** We retrospectively reviewed all acute aortic dissections (type A and B) who underwent emergency aortic surgery at our institution between 1994 and 2022 and identified 13 patients with acute aortic dissection during pregnancy or directly postpartum. Mann–Whitney U and Fisher’s exact tests were used for statistical analysis. **Results:** Of the 13 included patients, 5 had a genetic syndrome. These patients were significantly younger at the time of dissection and at an earlier stage of pregnancy (second trimester). Even though operative and in-house mortality was zero, we lost one patient on postoperative day 14 due to rupture of the aortic root after transfer to another hospital. Survival of neonates was 77% including two aborted pregnancies. **Conclusions**: Surgical treatment of acute aortic dissection during pregnancy can be performed with excellent operative mortality for the mothers and satisfying survival of their neonates. In patients with genetic syndrome, dissection occurs during the early second trimester, whereas non-syndromic patients experience acute dissection in the late third trimester. Long-term follow-up is essential for timely re-intervention, if needed.

## 1. Introduction

Classification of aortic dissection depends on the entry site. If located proximal to the subclavian artery, it is classified as Stanford type A aortic dissection, whereas when the entry tear is located distal to the subclavian artery, it is a Stanford type B aortic dissection.

Acute aortic dissection type A (AADA) is a clinical catastrophe associated with high morbidity and mortality [1]. It is often times a complication of pre-existing, frequently undiagnosed, proximal aortic aneurysm disease. Other known risk factors are untreated arterial hypertension, the presence of a bicuspid aortic valve, or known hereditary genetic syndromes (e.g., Marfan, Ehler–Danlos, Loeys–Dietz) recently resulting in a downward shift in the cutoff diameter for preemptive surgery in the current guidelines [2]. 

Acute aortic dissection type B (AADB) can often be treated conservatively by medical treatment; however, it can also be complicated by organ malperfusion, rupture or refractory pain or hypertension and treated by open surgery or—in most cases—endovascular therapy. An analysis of the International Registry of Acute Aortic Dissection reported a significantly lower in-hospital death rate in patients treated by endovascular repair compared to open surgery (11 vs. 34%, *p* = 0.002) [3].

Aortic dissection during pregnancy is direfully putting even two lives on the line and is potentially triggered by hormonal changes inducing alterations in the tunica media of the aorta [4] and increased circulating blood volume and cardiac output leading to elevated hemodynamic stress on the aortic vessel wall [5]. It is underestimated as the third most common cause of maternal death in the cardiovascular field [6] and 50% of AADA in women between 20 and 45 years of age are pregnancy-associated [7]. The incidence of AADA during pregnancy and puerperium has been steadily increasing over the past decades with 0.69 aortic dissections per 100.000 pregnancy-related hospitalizations [8]. Especially the third trimester and the postpartum period is the most vulnerable time interval as more than three-quarters of AADA occur in this period [9]. Maternal and fetal mortality have previously been described as between 35 and 53% [7,9]. Up to now, most case reports and, very rarely, larger series have been published of pregnant women with AADA [7]. One of the up-to-now largest series was published by Braverman et al., including 29 women (13 AADA, 16 AADB) of the IRAD [2]. We aimed for a larger analysis of characteristics and outcomes of AADA cases during pregnancy receiving emergency aortic surgery at our institution during the past 28 years.

## 2. Patients and Methods

### 2.1. Patient Selection

**Figure 1:**
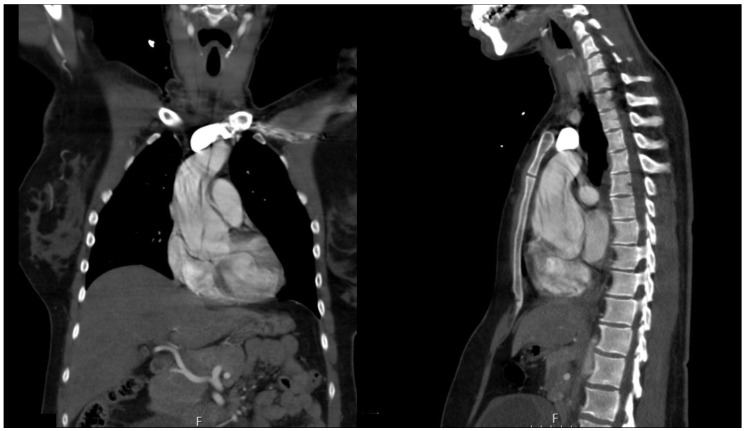
CT imaging with contrast agent of a pregnant woman, 27th gestational week.

**Figure 2:**
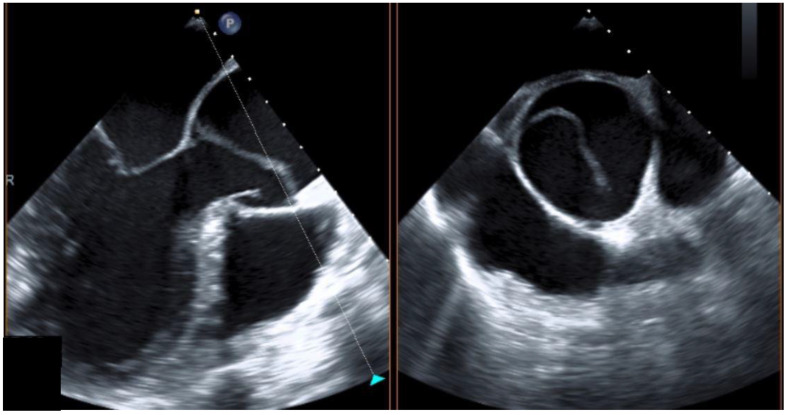
Transesophageal echocardiography in a pregnant woman with acute aortic dissection type A in the 38th gestational week.

The study was approved and informed consent was waived, by the ethics committee of the University of Leipzig (approval No 319-15-240802015). A retrospective analysis of our institutional database was performed evaluating all patients with acute aortic dissection type A and B between 1 January 1994 and 30 April 2022. Of a total of 1172 referred AADA patients, 13 were pregnant at the time of AADA or had just given birth (postpartum within 14 days). We also included one pregnant patient presenting with AADB. Patient charts, surgical reports, and imaging data (exemplarily CT imaging of AADA shown in Figure 1 and transesophageal echocardiography shown in Figure 2) were reviewed by one examiner. The extent of AADA was classified according to the classic Stanford classification.

### 2.2. Operative Technique

All pregnant AADA patients were treated in collaboration with the Department for Obstetrics and the Department for Pediatric Cardiology. In 8 cases a caesarean section was performed under general anesthesia directly prior to emergency aortic surgery. In three cases, AADA occurred postpartum and, in one case, a curettage was performed before aortic repair (Marfan patients with rapidly progressing type B aortic dissection). Extent of aortic surgery was determined by entry location and extent of dissection. In case of involvement of the aortic valve and root, either a combined replacement according to Bentall and de Bono [10] or a valve-sparing root replacement operation [11] was done. In case of arch involvement, patients received either a partial or total arch replacement [12] and, in the two cases with involvement of the thoracoabdominal aorta (TAA), a TAA repair was performed [13]. All surgical techniques have been previously published in detail.

### 2.3. Follow-Up

Follow-up was performed according to clinical routine protocol. Patients were routinely seen either in the aortic clinic or in the consultation hour for patients with known genetic syndromes.

### 2.4. Statistical Analysis

Categorical variables are presented as total numbers (percentages), and continuous variables are presented as mean with standard deviation and median. Statistical analysis was performed using SPSS version 29.0. Mann–Whitney U test was used for comparative analysis of continuous data and Fisher’s exact test was used for categorical data. Statistical significance is indicated by a *p*-value of <0.05.

## 3. Results

### 3.1. Patient Demographics

A total of 13 patients were analyzed, including 12 with AADA and 1 patient with AADB and rapidly progressing diameter (4 mm within two weeks). At the time of aortic dissection, the mean age of patients was 33.9 ± 5 years (range: 24–40), and patients with genetic syndrome were 6 years younger at the time of AADA (*p* = 0.02), for details see Table 1. Four of the five patients with genetic disorders had Marfan syndrome and one patient presented with Ehler–Danlos syndrome. The median gestation week of all patients was 33.0 (range 11–39). A separate analysis of patients with genetic disorders displayed that aortic dissection occurred significantly earlier in pregnancy (20.8 ± 9 weeks) compared to patients with non-syndromic aortic dissection (34.8 ± 7 weeks, *p* = 0.03). Arterial hypertension was the main co-morbidity, present in three out of five (60%) of patients with genetic syndrome and four out of five (88%) of spontaneous aortic dissection patients. All other co-morbidities were neglectably rare, and no relevant differences were present between the groups. Preoperative malperfusion was not different between the groups. 

### 3.2. Intraoperative Data

Aortic root repair was required in three out of eight non-syndromic patients and one out of five patients with genetic syndrome. However, the majority of patients with genetic syndrome required either repair (40%) or replacement (40%) of the aortic valve due to severe aortic valve regurgitation. In the non-syndromic group, the aortic valve was only affected in 50% of patients. 

The ascending aorta was replaced in all patients with non-syndromic AADA and 90% of the patients in the group of patients with genetic syndrome (including two patients with Type B dissection). The rate of partial arch replacement was significantly higher in the non-syndromic group of patients (86 vs. 20%, *p* = 0.03). Procedural times were similar between the two groups with a mean total surgical time of 301 ± 140 min vs. 297 ± 54 min, mean myocardial ischemia time of 82 ± 63 min vs. 103 ± 35 min, and mean circulatory arrest times of 33 ± 21 vs. 23 ± 9 min; for details see Table 2. Emergency thoracoabdominal aortic replacement was required in two patients with genetic syndromes.

### 3.3. Peri- and Postoperative Complications

Overall, peri- and postoperative complications occurred rarely. In each group, two patients had to be re-operated due to postoperative bleeding. Respiratory failure was seen in two patients with genetic syndrome and one with non-syndromic aortic dissection. Stroke and paraplegia affected one patient within each group; for details see Table 3. The sole patient with Ehler–Danlos syndrome type IV died within 30 days after surgery due to rupture of the aortic root after transfer to another hospital on POD 13, all other patients survived. Survival of neonates was 60% in the group with genetic syndrome and 88% in non-syndromic patients. A total of three patients required long-term reoperation: one Marfan patient underwent combined aortic valve and root replacement with a biological conduit and ascending replacement for aortic valve regurgitation and pseudoaneurysm of the ascending aorta after 5270 days and another Marfan patient received an ascending descending bypass due to stenosis of the ascending and arch prosthesis after 5213 days. In contrast, among non-syndromic patients, one was in need of a valve-sparing aortic root replacement, ascending replacement, aortic arch replacement with elephant trunk implantation after 265 days due to pseudoaneurysm of the aortic root, and intraprocedural dissection of the LAD immediately required coronary artery bypass grafting with a LIMA-LAD bypass. All patients survived their re-operations. 

## 4. Discussion

This retrospective single-center study included 13 patients with and without hereditary genetic syndrome affected by acute aortic dissection during pregnancy. We demonstrated that patients with genetic syndrome were significantly younger at the time of aortic dissection and experienced the dissection significantly earlier during pregnancy—while in the second trimester. 

Interestingly, patients without genetic syndrome significantly more often required partial arch replacement for either more distal entry or tear expansion [14]. The short- and long-term outcomes were not different between the groups with low 30-day mortality and a 25% overall rate of aortic reoperations during follow-up.

AADA is the most fatal aortic catastrophe known to be associated with high morbidity and mortality. Timely diagnosis and the presence of end-organ malperfusion are key determinants of survival. During pregnancy, AADA is scarce, and diagnosis in pregnant women is even more difficult due to incorrect assignment of symptoms in these young female patients, potentially leading to misdiagnosis and delayed initiation of the correct treatment path. Furthermore, limitations in imaging modalities (avoidance of X-ray/CT) aggravate diagnosis and also surgical planning for emergency aortic surgery. Not many large case series have been published on surgical outcomes of AADA in pregnant women. One larger series by Thalmann et al. included hospitalized and non-hospitalized female patients between 15 and 45 years of age with AADA in the city of Vienna but with only two cases of AADA during pregnancy [7]. The therein reported overall AADA mortality was high, reaching 60%; however, the two pregnant women survived. Nevertheless, a very recent study from Japan identified AADA as the most frequent cause of maternal death [15]. The analysis included 452 maternal deaths, 44 due to cardiac disease. Within this group, 40.9% (*n* = 18) of patients died due to aortic dissection, of which three had pre-diagnosed connective tissue disorder, preexisting arteritis, or family history of sudden death. 

An analysis by Braverman et al. of 9707 patients (3341 women) with aortic dissection included in the IRAD identified 29 cases of aortic dissection during pregnancy or postpartum [2]. Of those patients, 45% presented with type A and 55% with type B aortic dissection. They found that 64% of AADA occurred in the third trimester, whereas 50% of AADB occurred postpartum. All AADA patients received surgical therapy immediately, whereas 10/16 AADB patients could be treated medically. In contrast to our analysis, Braverman et al. showed that AADA occurred during the third trimester in 66% of patients with Marfan syndrome, whereas in our study, patients with genetic syndrome presented with AADA significantly earlier during the course of pregnancy [2]. However, numbers were small in both series.

In our analysis, 30-day mortality was low with only one death. This is in line with a recent study by Wang et al. [16] demonstrating excellent outcomes in six patients who suffered AADA during pregnancy (including two Marfan patients) and received emergency aortic repair. The mean gestational period at the time of AADA in this series was 35.3 ± 2.7 weeks, comparable to our non-syndromic group, and survival was 100% at discharge. All fetuses were successfully delivered by prior cesarean section and survived [16].

Another study from the UK by Yates et al. in 2015 included 11 pregnant patients receiving aortic surgery for aortic root dilatation with aortic regurgitation, aortic valve stenosis, and one for AADA [17]. The median gestational age was 19 weeks and four patients had Marfan syndrome. Maternal mortality was zero, and eight healthy babies were born at term, but three pregnancies ended as intrauterine demise within 1 week of surgery [17]. Our current series included one patient only with AADA in gestational week 17 receiving emergency aortic repair; however, luckily, the fetus survived and was born healthy in the 35th gestational week. 

The current ACC/AHA guidelines declare as a class 1 recommendation for acute aortic dissection during pregnancy: (i) urgent aortic surgery with fetal monitoring during the first and second trimester (LOE C-LD), (ii) urgent cesarean delivery immediately followed by emergency aortic surgery during the third trimester (LOE C-LD), and (iii) medical therapy unless endovascular or surgical therapy is needed for acute complications for acute type B dissection (LOE C-EO) [2]. 

In our analysis, patients without a genetic disorder received arch replacement limited to the proximal aortic arch significantly more often, whereas 40% of those with genetic syndrome directly received a total arch replacement. This is in line with data published 10 years ago by Schoenhoff et al. [18] on 94 patients with Marfan syndrome with a follow-up over 16 years. The group could demonstrate that 33% of Marfan patients initially presenting with AADA (and receiving only a partial arch replacement) required a secondary total arch replacement due to the progress of the disease. Of note, this was not the case in elective aortic surgery of Marfan patients, in this group, secondary arch re-intervention was only required in 3% of patients [18].

Rylski and the GERAADA group analyzed gender-related differences in AADA analyzing 1234 women and 1246 men included in the German Registry of Acute Aortic Dissection type A (GERAADA). Their study demonstrated that 49% of female patients (and almost the same percentage of male patients) received a hemiarch replacement for their initial AADA emergency repair [19]. The even higher rate of arch involvement revealed in our analysis could possibly be explained by hormonal changes during pregnancy affecting the aortic wall in general (rather than the proximal root only), which is potentially leading to a more complex pattern of dissection with a more extended dissection tear or more distally located entry (and re-entry) sites. It is known that during pregnancy, an increase in hemodynamic burden (higher circulating blood volume, higher heart rate) is present with a rise in cardiac output of 30 to 50% [5]. Furthermore, systemic vascular resistance is lowered and the elasticity of the aortic wall is increased by hormonal changes, paving the way for a further (and possibly more rapid) extension of the dissection.

In our current study, we demonstrated that patients with hereditary genetic syndrome experience acute aortic dissection at a significantly earlier stage during pregnancy, to be precise, in their second trimester and, on average, 15 weeks earlier than non-syndromic patients. It is known that aortic diameter slightly increases during pregnancy in healthy mothers. In patients with arterial hypertension, the progression of aortic diameter usually occurs during the last weeks of pregnancy [20]. A recent study on 400 pregnant women also showed that mean arterial blood pressure first decreased between gestational weeks 11 and 18 and thereafter increased until term [21]. The rise in blood pressure, together with a pre-diseased aortic wall due to the Fibrillin-1 mutation in patients with Marfan syndrome could explain the earlier occurrence of AADA. In addition, the aorta was found to grow more rapidly during pregnancy in Marfan patients compared to an age-matched cohort [22]. A recent, large analysis from the United States analyzed 472 pregnancy-related hospitalizations due to aortic dissection and revealed several co-morbidities in a multivariate logistic regression analysis for the onset of aortic dissection during pregnancy and puerperium, in detail: Marfan syndrome (OR 3469.39; *p* < 0.001), primary hypertension (OR 13.18, *p* < 0.001), chronic kidney disease (OR 8.34, *p* = 0.02), preeclampsia/eclampsia (OR 3.72, *p* < 0.001), smoking (OR 3.18, *p* < 0.001), and alcohol use (OR 6.01, *p* = 0.02) [8].

Recently several studies have analyzed the mode of delivery in women with known cardiovascular disease. However, a retrospective Italian multicenter study [23] identified 175 women with heart disease and defined a composite adverse maternal outcome including major postpartum hemorrhage, thrombo-embolic or ischemic events, de novo arrhythmia, heart failure, endocarditis, aortic dissection, need for re-surgery, sepsis, and maternal death. This study demonstrates that a scheduled cesarean delivery did not improve maternal outcomes but, at the same time, was associated with worse perinatal outcomes for the child [23]. However, another recent study showed that there is an association between the duration of labor and adverse peripartum cardiac outcome (including AADA) in patients with congenital heart disease. Prolonged duration of labor, meaning ≥24 h is associated with an increased risk for an adverse peripartum cardiac outcome (OR 2.7), especially in patients that after that time underwent a cesarean delivery (OR 6.8) [24]. Cesarean delivery is also recommended by the current ACC/AHA guidelines for the diagnosis and management of aortic disease [2] for patients with a history of chronic dissection (COR 1, LOE C-EO) or patients with an aortic diameter of root and/or ascending of ≥4.5 cm (COR 2a, LOE C-EO). In patients with an aortopathy and diameter <4.0 cm, a vaginal delivery is recommended (COR 1, LOE C-EO). In our series, one patient with a known ascending aorta of 3.9 cm and positive family history for AADA originally planned for vaginal delivery had a spontaneous dissection in the 38th gestational week. Decision-making in all patients with aortic diameter < 4.5 cm remains difficult and risk stratification is highly individual. The current guidelines also recommend that in patients with an aortic aneurysm or at increased risk of aortic dissection, pregnancy should be managed by a multidisciplinary team and delivery should be planned in a hospital with the capability for emergency aortic repair (COR 2a, LOE C-EO) [2]. It is also recommended that pregnant patients with aortopathies receive guideline-directed treatment of hypertension (COR 1, LOE C-LD) and, in syndromic patients, beta-blocker therapy is recommended during pregnancy and postpartum (COR 1, LOE C-EO) [2]. 

### Limitations

First, the retrospective design constitutes the main limitation of our study as data can only be analyzed as documented. The number of patients is small due to the nature of the disease. The number of pregnant patients misdiagnosed or diagnosed too late for surgical treatment remains unknown. 

## 5. Conclusions

We hereby report the up-to-now largest series of emergency aortic surgery in pregnant women with and without hereditary genetic syndrome with acute aortic dissection. Close monitoring of patients with Marfan syndrome, positive family history, and known aortic dilatation, as recommended in the current ACC/AHA guidelines, is essential. Furthermore, as AADA in the setting of pregnancy is often misdiagnosed or recognized with a certain delay, education of patients with predisposing factors and their families is important to support early correct diagnosis in the event of AADA. In patients with Marfan syndrome, AADA is likely to occur during the second or third trimester, whereas spontaneous AADA in non-syndromic patients rather occurs during the final weeks of pregnancy and at an older age of the mother. Regular check-ups after aortic surgery for AADA in an aortic clinic are mandatory for timely re-intervention if needed.

## Figures and Tables

**Table 1:** Baseline characteristics of all pregnant AADA patients.

	All Patients (*n* = 13)	Genetic Syndrome (*n* = 5)	Non-Syndromic (*n* = 8)	*p*-Value
Age [years]				
mean (SD)	33.9 (4.8)	30.2 (4.7)	36.3 (3.4)	0.02
median	34	30	37	
Gestation week				
Mean (SD)	28.5 (10.7)	20.8 (9.4)	34.8 (7.2)	0.03
median	33.0	17	39	
Aortic dissection				
Type A *n* (%)	12 (92)	4 (80)	8 (100)	0.4
Type B *n* (%)	1 (8)	1 (20)	0 (0)	0.3
Malperfusion *n* (%)				
Coronary	1 (8)	0 (0)	1 (13)	
Cerebral	1 (8)	0 (0)	1 (13)	1.0
visceral	1 (8)	1 (20)	0 (0)	
extremities	1 (8)	0 (0)	1 (13)	
aHT *n*(%)	10 (77)	3 (60)	7 (88)	0.5
Smoking *n* (%)	1 (8)	0 (0)	1 (13)	1.0
HLP *n* (%)	1 (8)	0 (0)	1 (13)	1.0
Atrial fibrillation *n* (%)	2 (15)	1 (20)	1 (13)	1.0
Family history *n* (%)	1 (8)	0 (0)	1 (13)	1.0
BAV *n* (%)	2 (15)	0	2 (25)	0.5
Marfan syndrome *n* (%)	4 (31)	4 (80)	0 (0)	n.a.

aHT: arterial hypertension; HLP: hyperlipidemia; BAV: bicuspid aortic valve; n.a.: not applicable; SD: standard deviation.

**Table 2:** Intraoperative data.

Operative Data	Patients (*n* = 13)	Genetic Syndrome (*n* = 5)	Non-Syndromic (*n* = 8)	*p*-Value
Root repair *n* (%)	4 (31)	1 (20)	3 (43)	1.0
Bentall procedure *n* (%)	3 (23)	2 (40)	2 (29)	1.0
David/Yacoub procedure *n* (%)	4 (21)	2 (40)	2 (14)	1.0
Ascending aortic replacement *n* (%)	12 (92)	4 (80)	8 (100)	0.4
Partial arch replacement *n* (%)	8 (62)	1 (20)	7 (86)	0.03
Total arch replacement *n* (%)	3 (23)	2 (40)	1 (14)	0.5
Thoracoabdominal aortic repair	2 (15)	2 (40)	0 (0)	0.2
Total surgical time [min]				
mean (SD)	299 (60)	301 (140)	297 (54)	1.0
median	283	336	272	
Ischemia time [min]				
mean (SD)	95 (45)	82 (63)	103 (35)	0.3
median	107	55	108	
CA time [min]				
mean (SD)	26 (12)	33 (21)	23 (9)	0.4
median	22	28	21	
Lowest temperature [°C]				
mean (SD)	25 (4)	25 (6)	25 (3)	1.0
median	26	25	26	

**Table 3:** Peri- and postoperative complications.

Intraoperative Data	Patients (*n* = 13)	Genetic Syndrome (*n* = 5)	Non-Syndromic (*n* = 8)	*p*-Value
30-day mortality	1 (8)	1 (20)	0 (0)	0.4
Survival of neonates	10 (77)	3 (60)	7 (88)	1.0
Postoperative complications:				
respiratory failure	3 (23)	2 (40)	1 (13)	0.5
renal failure	4 (31)	1 (20)	3 (38)	1.0
stroke	2 (15)	1 (20)	1 (13)	1.0
paraplegia	2 (15)	1 (20)	1 (13)	0.5
Re-exploration for bleeding	4 (31)	2 (40)	2 (25)	1.0
Long-term Re-operation	3 (23)	2 (40)	1 (13)	0.5
Follow-Up [days]				
Mean (SD)	2995 (2766)	4857 (3725)	2064 (1761)	n.a.
median	3015	5147	1960	

n.a.: not applicable.

## Data Availability

The data underlying this article will be shared on reasonable request to the corresponding author.

## Abbreviations

AADA	acute aortic dissection type A
aHT	arterial hypertension
BAV	bicuspid aortic valve
COR	class of recommendation
HLP	hyperlipidemia
LAD	left anterior descending
LIMA	left internal mammary artery
LOE	level of evidence
OR	Odds ratio
POD	postoperative day
TAA	thoracoabdominal aorta
